# Fitting magnetic field gradient with Heisenberg-scaling accuracy

**DOI:** 10.1038/srep07390

**Published:** 2014-12-09

**Authors:** Yong-Liang Zhang, Huan Wang, Li Jing, Liang-Zhu Mu, Heng Fan

**Affiliations:** 1 School of Physics, Peking University, Beijing 100871, China; 2 School of Civil Engineering and Mechanics, Lanzhou University, Lanzhou 730000, China; 3 Institute of Physics, Chinese Academy of Sciences, Beijing 100190, China; 4 Collaborative Innovation Center of Quantum Matter, Beijing 100190, China

## Abstract

The linear function is possibly the simplest and the most used relation appearing in various areas of our world. A linear relation can be generally determined by the least square linear fitting (LSLF) method using several measured quantities depending on variables. This happens for such as detecting the gradient of a magnetic field. Here, we propose a quantum fitting scheme to estimate the magnetic field gradient with N-atom spins preparing in W state. Our scheme combines the quantum multi-parameter estimation and the least square linear fitting method to achieve the quantum Cramér-Rao bound (QCRB). We show that the estimated quantity achieves the Heisenberg-scaling accuracy. Our scheme of quantum metrology combined with data fitting provides a new method in fast high precision measurements.

Magnetometry is important for mineral exploration and probing moving magnetic objects. High precision magnetometry^1^,^2^,^3^,^4^,^5^,^6^,^7^,^8^,^9^,^10^,^11^,^12^,^13^,^14^,^15^,^16^ also has wide applications in modern sciences and technologies, such as in nuclear magnetic resonance (NMR)^17^, magnetic resonance imaging (MRI)^18^,^19^, biomedical science^20^ and quantum control^21^. In some cases, the quantity interested is not the absolute strength of magnetic field but its difference and gradient. A standard measuring instrument for determining the gradient is differential atom interferometry, which utilizes two completely polarized atomic ensembles. Recently, quantum-enhanced measurements of magnetic field gradient have been proposed^22^,^23^,^24^,^25^.

It is by now well established that quantum metrology has advantages in enhancing precision of estimation^26^ which is beyond the classical method. In quantum metrology, the general framework for precision bound of estimation has been proposed and developed in Refs. 27–34, which is based on Fisher information (FI) and Cramér-Rao inequality. The precision of estimation depends on the amount of resources employed in the scheme, which might be for instance the number *N* of identical probes (photons, atoms) or the energy of probing field. The standard quantum limit, a consequence of the central limit theorem for statistics, shows that the precision is proportional to 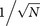. With quantum strategies such as entanglement and squeezing applied, one may attain better accuracy scaling as 1/*N*, which is the ultimate limit of precision named as Heisenberg limit. The NOON and GHZ states have been demonstrated to be able to provide a Heisenberg-limit sensitivity in some schemes^35^,^36^,^37^,^38^,^39^,^40^,^41^. Also some experiments have implemented the quantum enhanced metrology^42^,^43^,^44^,^45^,^46^,^47^.

In this work, we propose a quantum scheme of multi-parameter estimation to detect the gradient of magnetic field by employing *N*-atom spins. These atoms are initially prepared in W state, a genuine multipartite entangled state that can be generated in spin chain^48^ and has been experimentally produced by trapped ions^49^. These technologies can be utilized to implement our scheme in experiment. By applying the least square linear fitting method to the quantum enhanced multi-parameter estimation, we show that our scheme saturates the QCRB with Heisenberg-scaling accuracy. Let us highlight some advantages of this scheme: (i) Our scheme does not depend on the prior assumed linear assumption for the magnetic field, we essentially apply the reliable LSLF method. We also discuss that even if the linearity of the magnetic field is prior assumed, the bound of precision is exactly the same one. (ii) This simultaneous estimation scheme is in principle faster than repeated individual estimations. (iii) This is a general quantum fitting method and can be applied to measure other physical quantities with various fitting functions.

## Results

### Multi-parameter estimation combined with the least square linear fitting method

**Figure 1:**
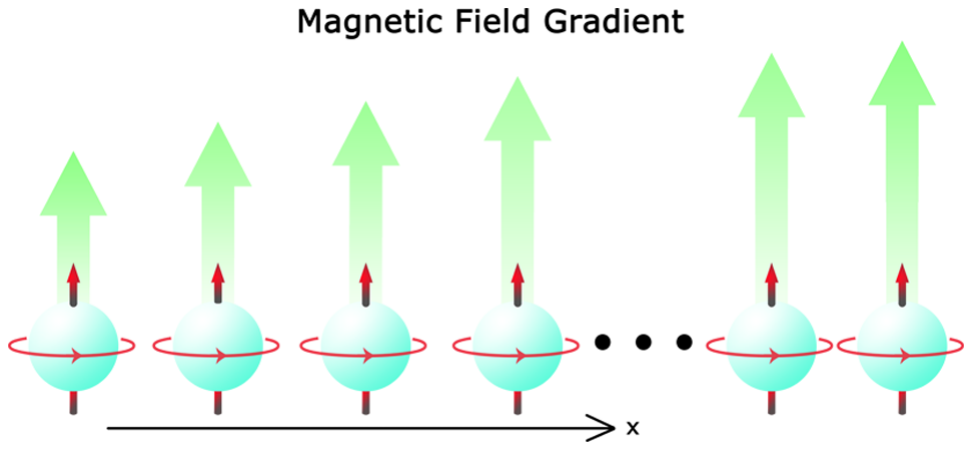
The schematic of the system. The atomic spin chain is coupled to a magnetic field, where each atom is separated with a distance *a* in the x-direction.

We consider the problem of measuring the gradient of a magnetic field. Our scheme is to simultaneously estimate the strength of magnetic field at different locations through quantum measurements and then to apply the LSLF method. We employ a *N*-atom spin chain as the probes, as shown in FIG. 1, to estimate the magnetic field gradient, where the *j*-th atom is located at *x_j_* = *x*_1_ + (*j* − 1)*a*, (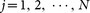) and the uncertainty of the location *x_j_* can be neglected. The Hamiltonian describes that each atom with two hyperfine spin states is coupled to the local magnetic field, and it takes the form, 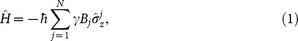
where *B_j_* and 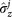 are the magnetic field and Pauli operator of atom *j*, and each atom has the same gyromagnetic ratio *γ*. The task of our scheme is to obtain optimal uncertainty bound of estimating the magnetic field gradient *G* that quantum mechanics permitted.

Initially, the atomic spins are prepared in a W state 
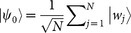
, where 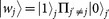. Considering that there are multiple independent parameters being estimated, they should be investigated in common ground. Then by symmetry consideration, W state is a good choice in multi-parameter estimation, which is largely different from single-parameter tasks. Further researches are needed to determine the best choice. For this closed quantum system, then the quantum state evolves under the action of magnetic field as 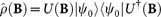, where 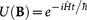 due to Schrödinger equaiton. The initial pure state acquired multiple phases through the unitary transformation is given by 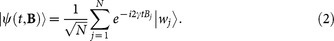
Because of an overall unobservable phase, it is proper to think that *B*_1_ = 0 always holds. Thus the covariance matrix **Cov**(**B**) and Fisher information matrix 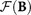 are size (*N* − 1) × (*N* − 1). Generalizing the expression of estimation for unitary dynamical processes^33^, the quantum Fisher information (QFI) matrix is given by 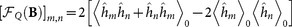
52, where 
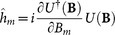
, 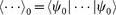. By straightforward calculations, one gets 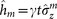, 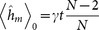 and 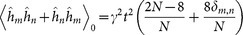, where *δ_m_*_,*n*_ is Kronecker's delta. Then the (*N* − 1) × (*N* − 1) sized QFI matrix and its inverse associated with the estimation of the magnetic field in our scheme is 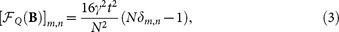

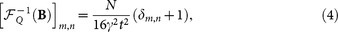
where *m*, 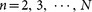.

Applying the LSLF method, we have the fitting gradient of the magnetic field as, 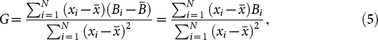
where 
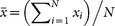
, 
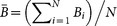
. Because each atom is separated with a distance *a* in the x-direction, then we get 
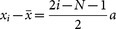
 and 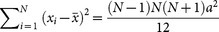. Thus the gradient of magnetic field is 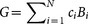, where the coefficients are 
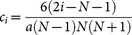
. Since the uncertainties of *x_j_* are neglected, the quantum Cramér-Rao inequality gives a lower bound on the variance of the magnetic field gradient 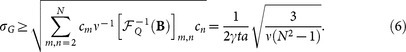
This bound is clearly a Heisengberg-scaling accuracy for large *N*. And the commutability of corresponding symmetric logarithmic derivatives (SLD) guarantees this bound can be saturated.

Now, we turn to the problem of constructing measurement strategy that can achieve quantum advantages in multi-parameter estimation. In this scheme, we construct two von Neumann measurement strategies, labeled by *a*, *b* respectively, 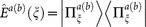, to be performed on the atomic spin chain as the following forms, 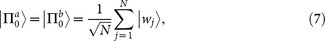

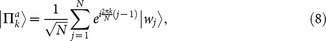

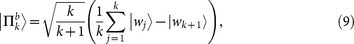
where 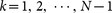. Both of these two sets of quantum states are orthonormal eigenstates of the coherence operator expressed as 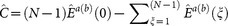, see Ref. 25. To implement these two von Neumann measurements in experiment, it requires performing global operators on N atoms instead of local operators on each atom. On the other hand, based on quantum theory, we can also first make a corresponding unitary transformation on those N atoms, then perform the local measurements in computational basis. These theoretical measurement strategies set a new goal for experimental physicists. By performing these operations 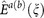, one obtains the ratio of each outcome 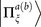, then determines the parameters *B_j_* through comparing these observed ratios with the probability distributions *p*(*ξ*|**B**). Based on the knowledge in the Methods section, one obtains the Fisher information matrices of these two measurement strategies, respectively, 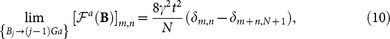

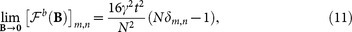
see supplementary material for detailed calculations. For strategy b, the limiting process **B** → 0 is equivalent to the small phases requirement 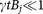 of local estimation theory in Method Section. For strategy a, we firstly choose the path *B_j_* = (*j* − 1)*Ga* to approach the limit **B** → 0. It's interesting that the Fisher information matrix has the same expression if one supposes that *B_j_* = (*j* − 1)*Ga*. So Eq. (10) takes the limit of *B_j_* → (*j* − 1)*Ga*.

For measurement strategy *a*, the Fisher information matrix is positive semi-definite and irreversible, which confirms that it is not an effective deterministic estimation. Applying Fourier transformation, we have 
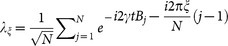
 and 
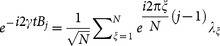
. This shows that the prerequisite for determining the magnetic field *B_j_* is knowing the module and argument of all *λ_ξ_*. Because the probability distributions associated with experimental outcomes are *p*(*ξ*|**B**) = |*λ_ξ_*|^2^/*N*, it is impossible to determine the argument of *λ_ξ_*. Thus this strategy is invalid for estimating magnetic field *B_j_*.

For measurement strategy *b*, which yields the QFI matrix, the probability of each outcome is transparently related to the magnetic field **B**, with *p*(1|**B**) involving only *B*_2_, *p*(2|**B**) involving only *B*_2_, *B*_3_, and so on^34^. Through comparing the ratio of observed measurement outcomes with the probability distributions, the estimator could sequentially determine the magnetic field 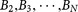. Then the gradient can be obtained by applying the LSLF method. Based on the results of asymptotically large *ν* independent experiments, this measurement strategy is optimal which can locally achieve quantum Cramér-Rao bound with Heisenberg-scaling accuracy. It is intriguing to explore how bad will be the degradation of this Heisenberg-scaling accuracy as some realistic imperfections kick in. Further researches are needed to conduct when one considers relevant imperfections like decoherence and particle losses.

### Single parameter estimation with linear assumption

If we assume that the magnetic field satisfies the linear condition *B_j_* = *B*_1_ + *G*(*j* − 1)*a*, the single parameter representing gradient *G* of magnetic field needs be estimated. In this case, the unitary transformation for the atomic spin chain is 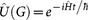, and the QFI can be expressed as^33^

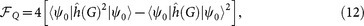
where 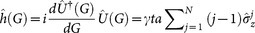. Applying this equation, we obtain 
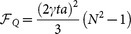
. It is straightforward to determine that the quantum Cramér-Rao bound 
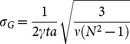
 which is exactly the same as the Heisenberg-scaling accuracy for scheme of the multi-parameter estimation. Immediately, we'll show that the previously proposed measurement strategies are optimal because they both yield the QFI and QCRB.

For measurement strategy *a*, its probability distributions and Fisher Information are 
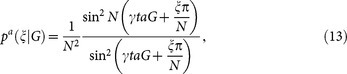

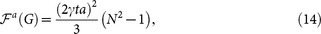
where the detailed calculations are showed in the supplementary material. The probability distribution *p^a^*(*ξ*|*G*) is clearly peaked around (−*ξ*/*N* + *j*)*π*/(*γta*) with approximate width *π*/(*Nγta*), where *j* is an arbitrary integer. If the condition 0 < *G* < *π*/(*γta*) is satisfied, one can successfully estimate *G* with Heisenberg-scaling accuracy. This measurement strategy is essentially a quantum Fourier algorithm for phase estimation^53^,^54^.

For measurement strategy *b*, we consider the estimation is local, i.e., the unknown parameter satisfies 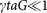. We show in the supplementary material that its Fisher information is 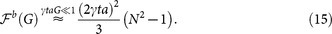
This implies that the Heisenberg-scaling QCRB can be reached locally via performing measurement strategy *b*.

## Discussion

Determining the gradient of magnetic field is inherently a multi-parameter estimation problem. We employ quantum enhanced multi-parameter estimation and the least square linear fitting method to achieve the Heisenberg-scaling quantum Cramér-Rao bound. Our scheme provides attainable high precision in magnetometry. This proposal is the first data fitting scheme possessing Heisenberg-scaling accuracy. This opens a new avenue for the investigations of general data fitting problems.

## Methods

Here, let us introduce the method used in this work. We next will present a brief review of local estimation theory, the Fisher information and Cramér-Rao inequality^27^,^28^,^29^,^30^,^31^,^32^,^33^.

Considering a curve 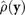 characterizing dynamical process on the space of density matrix, the problem of determining the value of the parameter vector 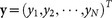 is a fundamental problem of statistical inference based on the experimental results. Before the measurements, we know that an observable random variable *ξ* carries information about the unknown parameter vector **y**, which is described by the smooth probability distribution *p*(*ξ*|**y**). The normalization is 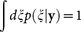, and *ξ* could be discrete or multivariate although it is written here as a single continuous real variable.

**Figure 2:**
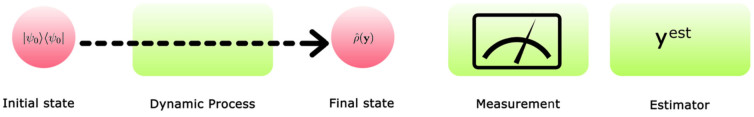
Scheme of quantum parameter estimation. The finial state 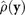, evolved from a known initial state allowed by quantum mechanics, carries about the parameter vector characterizing dynamical process, and **y***^est^* is obtained from the measurement results performed on the final state.

Then we take a random sample of size *ν* to estimate the parameter vector **y** via comparing the ratio of observed measurement outcomes with the probability distribution. An essential premise of effective deterministic estimation is requiring that the smooth map *p*(*ξ*|**y**) ↔ **y** is bijective. In order to avoid the periodical problems of determining the parameters *y_i_*, it is generally assumed that all components *y_i_* are small, which is called local estimation. For an effective deterministic observable random variable *ξ*, one estimates the parameter vector **y** via funtions 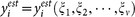 based on experimental results. The general framework of quantum parameter estimation is shown in FIG. 2. Then the expectation and covariance matrix of estimation are 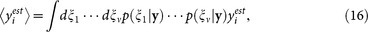

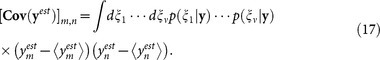


Taking the partial derivative of Eq.(16) with respect to *y_j_* and combining them into a bilinear quadratic form via two arbitrary real vectors 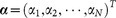, 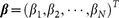, we obtain 
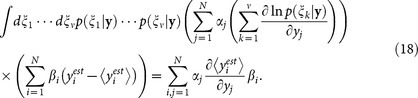
Applying the Cauchy-Schwarz inequality to Eq.(18) yields Cramér-Rao bound^27^,^28^,^29^,^30^,^31^,^32^

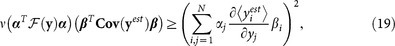
where the Fisher information (FI) matrix is defined by 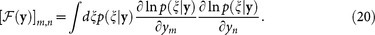
Based on Eq.(19), for all ***α***, there exits ***β*** s.t. 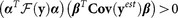, and because ***β****^T^* **Cov**(**y***^est^*)***β*** ≥ 0, then we find that the Fisher information matrix 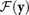 is positive. Noticing that Eq.(19) only holds for effective deterministic estimation, the Fisher information matrix defined by Eq.(20) is merely positive semi-definite for arbitrary observable random variables.

The asymptotic theory of maximum-likelihood estimation states that^27^,^31^,^32^, in the approximate sense for large *ν*, the estimation achieves the Cramér-Rao bound and is unbiased locally, i.e. 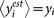, where **Cov**(**y***^est^*) is the matrix describing the deviation between the estimated values and real values. Thus for unbiased effective deterministic estimation, the Cramér-Rao inequality can be written as refs. 29, 32

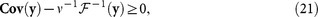
which means that it is a positive semi-definite matrix.

For quantum mechanics, the generalized measurement performed on the density matrix 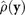 is described by a set of of non-negative Hermitian operators 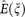
53, which are complete in the sense that 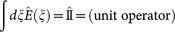. And the probability distribution for measurement outcomes *ξ* is given by 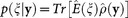. As proven in ref. 31, we have 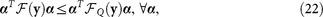
where 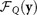 is the so-called quantum Fisher information (QFI) matrix defined as refs. 29, 30, 32

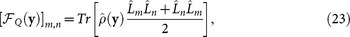
where these Hermitian operators are the so-called symmetric logarithmic derivatives (SLD), defined by the following equation 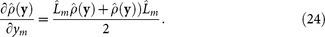
The sufficient and necessary conditions for equality holding in Eq.(22) are 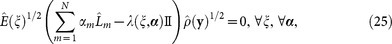
where 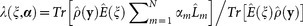 is real. For single parameter estimation, the equality in Eq.(22) can always be satisfied by choosing the Hermitian operators to be one-dimensional projectors onto a complete set of orthonormal eigenstates of 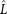
31. Thus quantum Fisher information is the maximum of Fisher information over all possible measurement strategies^31^,^33^, i.e. 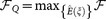. For multi-parameter estimation, the equality in Eq.(22) generally is not achievable, which means that the quantum Cramér-Rao inequality 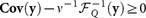 cannot always be saturated^29^,^30^,^32^,^34^,^50^,^51^,^52^. One obvious sufficient condition for the attainability of QCRB is the commutators of SLDs are zero.

## Author Contributions

Y.L.Z. and H.F. proposed this project, Y.L.Z. made the main calculations. H.W. and L.J. involved in analyzing the results and discussions. Y.L.Z. and H.F. wrote the paper with comments from all other authors. H.F. and L.Z.M. supervised the project.

## Supplementary Material

Supplementary Information Supplementary material
