# Supplementary material for: Fitting magnetic field gradient with Heisenberg-scaling accuracy
Source: Sci Rep. 2014 Dec 9;4:7390. doi: 10.1038/srep07390 (PMC4260217; doi:10.1038/srep07390)
Supplement: Supplementary Information — Supplementary material [file srep07390-s1.pdf]

Supplementary material: Fitting magnetic field gradient with Heisenberg-scaling accuracy

Yong-Liang Zhang,[1] Huan Wang,[2] Li Jing,[1] Liang-Zhu Mu*,[1] and Heng Fan†[3,4]
[1]*School of Physics, Peking University, Beijing 100871, China*
[2]*School of Civil Engineering and Mechanics, Lanzhou University, Lanzhou 730000, China*
[3]*Institute of Physics, Chinese Academy of Sciences, Beijing 100190, China*
[4]*Collaborative Innovation Center of Quantum Matter, Beijing, China*
(Dated: August 18, 2014)

Here, we will provide some detailed calculations.

I. CALCULATION OF FISHER INFORMATION MATRIXES IN MULTI-PARAMETER ESTIMATION

For von Neumann measurements $|\Pi_\xi\rangle = \sum_{j=1}^{N} U_{(\xi+1),j}|w_j\rangle$, the probability distribution of each measurement result is

$$p(\xi|\mathbf{B}) = Tr[\hat{E}(\xi)\hat{\rho}(\mathbf{B})] = \sum_{\mu,\nu=1}^{N} z_\mu \widetilde{z}_\nu U_{(\xi+1),\mu}\widetilde{U}_{(\xi+1),\nu}, \tag{1}$$

where $z_\mu = \frac{1}{\sqrt{N}}e^{i2\gamma t B_\mu}$, $\widetilde{z}$ denote the complex conjugate of z and U is a unitary matrix, i.e., $\sum_{k=1}^{N} U_{k,\mu}\widetilde{U}_{k,\nu} = \delta_{\mu,\nu}$. Then the Fisher information matrix is

$$[\boldsymbol{\mathcal{F}}(\mathbf{B})]_{m,n} = \sum_{\xi=0}^{N-1} \frac{\partial_{B_m} p(\xi|\mathbf{B})\partial_{B_n} p(\xi|\mathbf{B})}{p(\xi|\mathbf{B})} \tag{2}$$

$$= 4\gamma^2 t^2 \sum_{k=0}^{N} \frac{\sum_{\mu,\nu=1}^{N}[-iz_\mu\widetilde{z}_m U_{k,\mu}\widetilde{U}_{k,m} + i\widetilde{z}_\mu z_m \widetilde{U}_{k,\mu}U_{k,m}] \times [-iz_\nu\widetilde{z}_n U_{k,\nu}\widetilde{U}_{k,n} + i\widetilde{z}_\nu z_n\widetilde{U}_{k,\nu}U_{k,n}]}{\sum_{\mu,\nu=1}^{N} z_\mu\widetilde{z}_\nu U_{k,\mu}\widetilde{U}_{k,\nu}} \tag{3}$$

$$= 4\gamma^2 t^2 \sum_{k=1}^{N} 2Re\Big[\widetilde{z}_m z_n \widetilde{U}_{k,m}U_{k,n} - \widetilde{z}_m\widetilde{z}_n\widetilde{U}_{k,m}\widetilde{U}_{k,n}\frac{\sum_{\mu,\nu=1}^{N} z_\mu z_\nu U_{k,\mu}U_{k,\nu}}{\sum_{\mu,\nu=1}^{N} z_\mu\widetilde{z}_\nu U_{k,\mu}\widetilde{U}_{k,\nu}}\Big] \tag{4}$$

$$= 8\gamma^2 t^2 \Big[\frac{1}{N}\delta_{m,n} - Re\Big(\widetilde{z}_m\widetilde{z}_n \sum_{k=1}^{N}\widetilde{U}_{k,m}\widetilde{U}_{k,n}\frac{\sum_{\mu,\nu=1}^{N} z_\mu z_\nu U_{k,\mu}U_{k,\nu}}{\sum_{\mu,\nu=1}^{N} z_\mu\widetilde{z}_\nu U_{k,\mu}\widetilde{U}_{k,\nu}}\Big)\Big]. \tag{5}$$

For measurements $\hat{E}^a(\xi) = |\Pi^a_\xi\rangle\langle\Pi^a_\xi|$, the unitary matrix is $U^a_{\mu,\nu} = \frac{1}{\sqrt{N}}e^{\frac{i2\pi(\mu-1)(\nu-1)}{N}}$. If one supposes that $B_j = (j-1)aG$, then

$$\sum_{\mu=1}^{N} z_\mu U_{k,\mu} = \frac{\sin N(\gamma taG + \frac{(k-1)\pi}{N})}{N\sin(\gamma taG + \frac{(k-1)\pi}{N})} e^{i(N-1)(\gamma taG + \frac{(k-1)\pi}{N})}, \tag{6}$$

$$\lim_{\{B_j\to(j-1)Ga\}} [\boldsymbol{\mathcal{F}}^a(\mathbf{B})]_{m,n} = \frac{8\gamma^2 t^2}{N}\Big[\delta_{m,n} - \frac{1}{N}Re\Big(e^{-i2\gamma taG(m+n-N-1)}\sum_{k=1}^{N} e^{-\frac{i2\pi(k-1)}{N}(m+n-N-1)}\Big)\Big] \tag{7}$$

$$= \frac{8\gamma^2 t^2}{N}(\delta_{m,n} - \delta_{m+n,N+1}) \tag{8}$$

* muliangzhu@pku.edu.cn
† hfan@iphy.ac.cn

For measurements $\hat{E}^b(\xi) = |\Pi^b_\xi\rangle\langle\Pi^b_\xi|$, the unitary matrix degrades into an orthogonal matrix $O^b_{\mu,\nu}$, we have

$$\lim_{\mathbf{B}\to\mathbf{0}} \frac{\sum_{\mu,\nu=1}^{N} z_\mu z_\nu O^b_{k,\mu} O^b_{k,\nu}}{\sum_{\mu,\nu=1}^{N} z_\mu \widetilde{z}_\nu O^b_{k,\mu} O^b_{k,\nu}} = 2\delta_{k,1} - 1, \tag{9}$$

$$\lim_{\mathbf{B}\to\mathbf{0}} [\boldsymbol{\mathcal{F}}^b(\mathbf{B})]_{m,n} = \frac{16\gamma^2 t^2}{N^2}(N\delta_{m,n} - 1). \tag{10}$$

II. CALCULATION OF FISHER INFORMATION IN SINGLE PARAMETER ESTIMATION

Since $\frac{\sin N(\gamma taG + \frac{\xi\pi}{N})}{\sin(\gamma taG + \frac{\xi\pi}{N})} = \sum_{m=-J}^{J} e^{i2m(\gamma ta + \frac{\xi\pi}{N})}$, where $J = (N-1)/2$, we calculate the probability distribution and the Fisher information of measurements $\hat{E}^a(\xi) = |\Pi^a_\xi\rangle\langle\Pi^a_\xi|$

$$p^a(\xi|G) = \frac{1}{N^2}|\sum_{j=1}^{N} e^{-i2(j-1)(\gamma taG + \frac{\xi\pi}{N})}|^2 = \frac{1}{N^2}\frac{\sin^2 N(\gamma taG + \frac{\xi\pi}{N})}{\sin^2(\gamma taG + \frac{\xi\pi}{N})}, \tag{11}$$

$$\mathcal{F}^a(G) = \sum_{\xi=0}^{N-1} \frac{1}{p^a(\xi|G)}[\frac{dp^a(\xi|G)}{dG}]^2 = \frac{4}{N^2}\sum_{\xi=0}^{N-1}[\frac{d}{dG}\frac{\sin N(\gamma taG + \frac{\xi\pi}{N})}{\sin(\gamma taG + \frac{\xi\pi}{N})}]^2 \tag{12}$$

$$= \frac{(2\gamma ta)^2}{N^2}\sum_{\xi=0}^{N-1}\sum_{m,m'=-J}^{J} 4mm' e^{i2(m-m')x + i2(m-m')\frac{\xi\pi}{N}}$$

$$= \frac{(2\gamma ta)^2}{N^2}\sum_{m,m'=-J}^{J} 4mm' e^{i2(m-m')x} N\delta_{m,m'} = \frac{(2\gamma ta)^2}{3}(N^2-1). \tag{13}$$

The probability distribution and the Fisher information of measurements $\hat{E}^b(\xi) = |\Pi^b_\xi\rangle\langle\Pi^b_\xi|$ are

$$p^b(0|G) = \frac{1}{N^2}\frac{\sin^2(N\gamma taG)}{\sin^2(\gamma taG)} \overset{\gamma taG\ll 1}{\approx} 1 + \frac{(N^2-1)(\gamma taG)^2}{3}, \tag{14}$$

$$p^b(\xi|G) = \frac{\xi}{N(\xi+1)}\Big[1 - 2\cos[(\xi+1)\gamma taG]\frac{\sin(\xi\gamma taG)}{\xi\sin(\gamma taG)} + \Big(\frac{\sin(\xi\gamma taG)}{\xi\sin(\gamma taG)}\Big)^2\Big] \overset{\gamma taG\ll 1}{\approx} \frac{\xi(1+\xi)(\gamma taG)^2}{N}, \tag{15}$$

$$\mathcal{F}^b(G) = \sum_{\xi=0}^{N-1}\frac{1}{p^b(\xi|G)}[\frac{dp^b(\xi|G)}{dG}]^2 \overset{\gamma taG\ll 1}{\approx} \frac{(2\gamma ta)^2}{3}(N^2-1) \tag{16}$$
